# Modeling and prediction of pressure injury in hospitalized patients using artificial intelligence

**DOI:** 10.1186/s12911-021-01608-5

**Published:** 2021-08-30

**Authors:** Christine Anderson, Zerihun Bekele, Yongkai Qiu, Dana Tschannen, Ivo D. Dinov

**Affiliations:** 1 School of Nursing, University of Michigan, Ann Arbor, MI 48109 USA; 2 Statistics Online Computational Resource (SOCR), University of Michigan, Ann Arbor, MI 48109 USA; 3 Department of Applied and Computational Mathematics and Statistics, University of Notre Dame, Notre Dame, IN 46556 USA; 4 Department of Health Behavior and Biological Sciences (HBBS), School of Nursing, University of Michigan, Ann Arbor, MI 48109 USA; 5 Michigan Institute for Data Science (MIDAS), University of Michigan, Ann Arbor, MI 48109 USA

**Keywords:** Health analytics, Precision medicine, Clinical assessment, Human–machine intelligence

## Abstract

**Background:**

Hospital-acquired pressure injuries (PIs) induce significant patient suffering, inflate healthcare costs, and increase clinical co-morbidities. PIs are mostly due to bed-immobility, sensory impairment, bed positioning, and length of hospital stay. In this study, we use electronic health records and administrative data to examine the contributing factors to PI development using artificial intelligence (AI).

**Methods:**

We used advanced data science techniques to first preprocess the data and then train machine learning classifiers to predict the probability of developing PIs. The AI training was based on large, incongruent, incomplete, heterogeneous, and time-varying data of hospitalized patients. Both model-based statistical methods and model-free AI strategies were used to forecast PI outcomes and determine the salient features that are highly predictive of the outcomes.

**Results:**

Our findings reveal that PI prediction by model-free techniques outperform model-based forecasts. The performance of all AI methods is improved by rebalancing the training data and by including the Braden in the model learning phase. Compared to neural networks and linear modeling, with and without rebalancing or using Braden scores, Random forest consistently generated the optimal PI forecasts.

**Conclusions:**

AI techniques show promise to automatically identify patients at risk for hospital acquired PIs in different surgical services. Our PI prediction model provide a first generation of AI guidance to prescreen patients at risk for developing PIs.

**Clinical impact:**

This study provides a foundation for designing, implementing, and assessing novel interventions addressing specific healthcare needs. Specifically, this approach allows examining the impact of various dynamic, personalized, and clinical-environment effects on PI prevention for hospital patients receiving care from various surgical services.

**Supplementary Information:**

The online version contains supplementary material available at 10.1186/s12911-021-01608-5.

## Background

Pressure injuries (PIs) continue to negatively impact clinical practice and increase patient suffering. The enduring incidence of PIs in hospitals and other healthcare settings frustrate all stakeholders, including patients, families, caregivers, insurers, and health-policymakers. The expectation is that providers can thwart these “preventable” injuries. However, due to the many potential clinical dimensions that increase the risk for developing PIs, and the dynamic nature of the problem, they persist.

Research reports in the literature about the incidence and prevalence of PIs are numerous. Three recent systematic reviews illustrate the difficulty of quantifying the incidence and prevalence of the problem.

Bufone et al*.* [1] conducted a systematic review of perioperative PIs. Of the eleven articles that met their inclusion criteria, the incidence range was between 1.3% and 54.8%. Although a meta-analysis was not performed, the range in reported outcomes was highly variable. The authors note that likely contributors to their heterogeneous findings were that the studies varied on the PI stages that were included (e.g., Stage I, I & II, III & IV), assessment tool used (Braden, Norton, RAPS or not reported), and type of surgery (orthopedics, cardiac, ENT, others). Recommendations for future research included clarifying the differences in PI among different surgical services and using risk assessment tools that include intraoperative variables [1].

Jackson et al*.* [2] reviewed 29 cross sectional and cohort studies about the prevalence and incidence of PI related to medical devices. The studies included all age groups, mixed unit types, and took place in 14 countries with data from 126,150 patients. The pooled incidence reported in 13 studies was 12% (95% CI 8–18) with heterogeneity of *I*^2^ = 95.9%, *p* < 0.001. The pooled prevalence from 16 studies was 10% (95% CI 6–16). Many of the PI involved mucosal tissue, so the stage was not provided in most of the included studies. Explanations for the heterogeneity included variation in clinical environments, patient characteristics, types of devices and staging [2].

Chaboyer et al*.* [3] reviewed 22 observational, cross sectional and cohort studies of adult patients in intensive care units (ICUs). The findings from the meta-analysis were a pooled incidence range (95% CI 10.0 – 25.9), *I*^2^ = 98 and pooled prevalence (95% CI 16.9 – 23.8), *I*^2^ = 92. High heterogeneity was explained by variation in measurement methods, regional variation and a range of data that may have been contributory but was not provided, for example delivery of PI prevention strategies, nurse/patient ratios and length of stay in the ICU.

These reviews, encompassing 62 studies, illustrate two primary conclusions: First, the incidence and prevalence of PI remain unacceptably high; and second, there is widespread heterogeneity in epidemiological studies of PI. Reducing the problem of PI is critically important. The purpose of this research was to develop accurate PI prediction models that can be translated into practical tools for individualized and targeted PI prevention interventions. Specifically, the study goals were to:

1. Build model-based statistical inference and model-free artificial intelligence (AI) techniques to mine and interrogate high-dimensional clinical data aiming to:
  - predict specific PI related clinical outcomes for patients:
  - identify salient features in the data that are highly predictive of the outcomes, and
2. Derive computed phenotypes by unsupervised clustering.

### Risk prediction complexity and Pressure Injury Prediction Modeling (PIPM)

In order to reduce the incidence and prevalence of PI, prevention is critical. Risk assessment is at the forefront of this effort and has been the subject of research for decades. Tschannen and Anderson’s [4] synthesis of existing PI conceptual models showed that despite the extensive literature on the topic, gaps remain in understanding PI risk. The authors reviewed 59 studies that showed current evidence about factors that predict hospital acquired PIs. After synthesizing the evidence, the Pressure Injury Prediction Model (PIPM) was developed representing 6 constructs: *pressure, tissue tolerance, friction and shear, as well as three new constructs—patient characteristics, environment, and episode of care*. These constructs arose from 53 concepts, often having multiple measures and indicators. Similar to the incidence and prevalence studies, the sheer number of possible predictors and predictor combinations contributes to the complexity of PI risk assessment and prevention [4]. The advancement of the electronic health record (EHR) provides the opportunity for large scale data analysis, accounting for all of the identified predictors of hospital-acquired PIs.

### PI risk prediction using EHR

Widespread use of the electronic health record (EHR) has led to the collection of vast amounts of clinical data. As a result, researchers are developing new methods to improve PI prediction that take advantage of this both in terms of the number of cases analyzed and in the granularity of the variables used. For example, Rondinelli et al*.* [5] conducted a retrospective cohort study of over 700,000 inpatient episodes in 35 hospitals to examine the time from admission to the development of a healthcare acquired PI. Independent variables included age, gender, diagnoses, admission and discharge information and comorbidities that were present on admission. A comorbidity point score, severity of illness score and the overall Braden Scale were also used. Their analysis, using a multivariate Cox proportional hazards model showed significant hazard ratios for age, severity of illness, comorbidity and the Braden scale as risk predictors. Significant variation among the hospitals was also found.

Using another approach, Jin et al*.* [6] used multiple logistic regression to select variables extracted from the EHR and to develop an algorithm for PI risk prediction that was then tested in real time. Ten factors, from the original 4,211, resulted in a daily risk score that compared favorably with the Braden score, but did not require any input from the nursing staff. The researchers noted that the risk score did not allow the staff to view specific risk factors, nor did it account for any injury prevention interventions.

Recent analytic approaches to identifying PI risk more accurately has included the use of advanced data science analytics with Big Data. Advanced analytic methods, such as machine learning and artificial intelligence (AI) techniques, allow analysis of large, incongruent, incomplete, heterogeneous, and time-varying data [7–11]. More simply, the use of machine learning allows researchers to look for patterns that can help explain the current state or be used to make predictions about the future [12]. Such data analysis techniques have been used successfully in determining predictors of other health outcomes, such as catheter associated urinary tract infection [13], septic shock [14], Parkinson’s disease [15], and more recently, PIs.

Recent efforts to predict PIs more accurately have included the use of data science analytics and machine learning. For example, Kaewprag and colleagues [16] evaluated 7,717 ICU patient records (590 patients with PI) using 6 machine learning algorithms to develop predictive models for PI. Their methods included first univariate analysis to determine association and then logistic regression, support vector machine, decision tree, random forest, k-nearest neighbor and Naïve Bayes to analyze data that included variables associated with the Braden Scale, medications, and diagnosis. Logistic regression and Naïve Bayes models yielded the highest area under the receiver operating characteristic curve (AUC). Specifically, the combination of diagnosis and Braden features yielded the best predictive model for PI incidence, with an AUC using logistic regression of 0.83 compared with the Braden features alone at 0.73, however the sensitivity was low at 0.160.The Naïve Bayes method had a lower AUC (0.815) and better sensitivity (0.628).Although the study integrated over 828 unique medications and 861 diagnoses, other contributing factors for PI incidence were not included, thus potentially limiting the overall accuracy of the risk predictor.

In another study, Hu et al. [17] created three prediction models for inpatient PI using machine learning techniques (e.g. decision tree, logistic regression, and random forest). Analysis of 11,838 inpatient records—including both indirect and direct variables of interest—found 36 significant predictors of PI development. Attribute selection was initially based on correlation analysis prior to model development. The model built using random forest was the strongest, with precision of 0.998, and the average AUC of 1.00, in the training set, however the AUC in the validation set the AUC was 0.864 with random forest still providing the best results. Although much more inclusive of previously identified predictors of PI, exclusions of key variables (e.g. presence of comorbidities, oxygenation and/or nutrition deficits, friction and shear) were noted. In addition, a very limited sample of patients with a PI (1.5% of sample) was included, which may have contributed to the difference between training and validation sets (e.g. treatment of the outcome imbalance in the training sets).

Cramer and colleagues [18] examined structured EHR data from 50,851 admissions to predict PI in the ICU using various machine learning techniques. Models that incorporated over 40 EHR features were captured in the dataset, accounting for the first 24 h of admission, including physiologic, admission, and lab variables. Analysis was conducted on training and test sets using advanced techniques such as logistic regression, elastic net, support vector machine, random forest, gradient boosting machine, and a feed forward neural network approach. Findings identified the weighted logistic regression model to be the best model, although all models were limited in their precision (0.09–0.67) and recall (0–0.94). Despite this, the model outperformed the Braden scale, the traditional approach to identifying risk for PI. Similar to other studies, imbalance in the sample and missing data were identified as limitations in the analysis.

Despite the promising use of advanced data analytics in determining the true risk for PI development, several limitations in the work to date require further exploration. Several of the studies conducted using machine learning thus far have shown improved risk prediction [17, 19, 20]. However, prior reports also use limited model features, sample imbalances, and missing data as limitations. For this reason, further exploration with a large dataset incorporating all predictors of PI risk is needed.

More broadly, over the past decade a number of powerful computational and artificial intelligence approaches have been developed, tested, and validated on medical data [21–23]. Most techniques have advantages and limitations. For instance, some evidence suggests that Support Vector Machine (SVM) and Artificial Immune Recognition System (AIRS) are very reliable in specific medical applications [21]. Whereas deep neural network learning yields the most statistically consistent, accurate, reliable, and unbiased results in medical image classification, parcellation, and pathological detection [24, 25].

## Methods

### Data

Using the PIPM as a guide, we extracted two years of clinical and administrative data from a large, tertiary health system. Electronic health record (EHR) data for over 23,000 patient encounters discharged between June 1^st^, 2014 and June 26^th^, 2016 were obtained from the study site. The health system uses the EPIC^©^ vendor for inpatient documentation. Inclusion criteria for extraction of patient records included: [1] adults (≥ 18 years of age); [2] undergoing a surgical procedure; and [3] hospitalized for two or more days between June 2014 and June 2016. Administrative data, including nurse staffing data, were extracted for all nursing units where the patients were admitted during the study timeframe.

Specific data elements extracted for the study aligned with the PIPM framework. PIPM represents the many risk factors for PI that have been reported in the literature, and organized into six constructs: patient, pressure, shear, tissue tolerance, environment and episode of care. Each construct includes multiple concepts that are in turn, represented by indicators and measures that were used to develop the data dictionary and the extraction plan.

Staffing level data were collected for each day the patient spent on specific patient care units. Likewise, data pertaining to the intraoperative phase were collected for each procedure that occurred during the specific hospitalization. For example, the American Society of Anesthesiologists (ASA) score was used as a measure of severity of illness and the length and type of the procedure were recorded for each operation.

All data was initially captured as a raw csv file. Data was cleaned and formatted for use with statistical software. The data were collected for each day the patient spent in the hospital and, when relevant, for each surgical procedure if more than one operation occurred. For example, age, gender and ethnicity were demographic features that did not change over the course of the hospital stay whereas vital signs, medications and Braden scores were collected for each day. For the purposes of this study, data were aggregated to hospital-stay level variables. For example, total Braden scores were captured for each day of a patient’s stay, respectively. These values were then aggregated to three stay-level variables: Total Braden min (e.g., lowest Braden score for the stay); Total Braden max (e.g., highest Braden score for the stay); and Total Braden average (e.g., average score of all daily Braden scores).

In the Additional file 1 section, we include a table mapping the compressed variable names into explicit clinically relevant descriptions.

### Data preprocessing

**Fig. 1:**
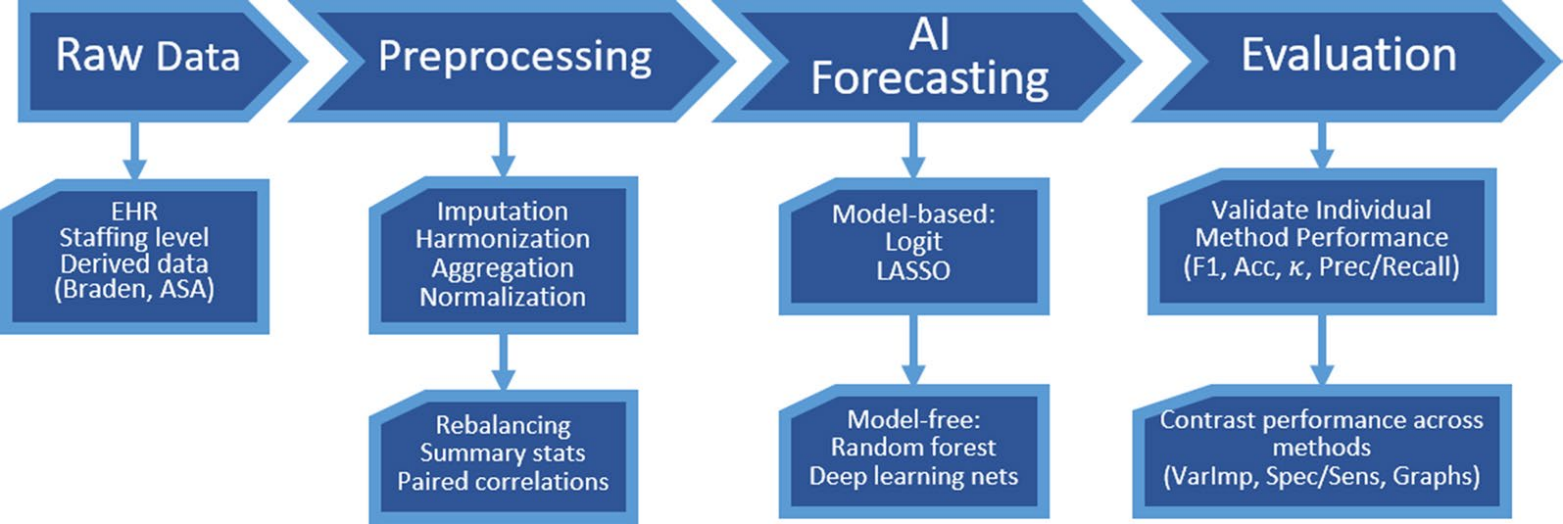
Graphical flowchart illustrating the end-to-end pipeline process from ingesting the raw data, through the preprocessing, modeling, analysis, prediction, and visualization of the results

We used previously developed and validated data preprocessing protocols [26–29]. These include imputation of missing values, data harmonization and aggregation across multiple data tables, hot-coding of categorical variables as numeric dummy variables, normalization to facilitate cross-feature distance calculations, rebalancing to stabilize the sample-sizes in different cohorts, and extraction of summary statistics characterizing the distributions of various features. Table 1 shows examples of preprocessing steps for different types of biomarkers. This preprocessing was necessary to generate an integrated canonical form of the data as a computable object that can be visualized, analyzed, models, and train the AI/ML classifiers. Figure 1 depicts the key elements of the end-to-end computational workflow we built to ingest the heterogeneous data, perform preprocessing, fit models and derive model-free prediction, and validate the performance of different methods.

**Table 1:** Illustrations of alternative types of data preprocessing filters applied to different types of clinical measurements

Item	Date group/type	Data type/nature	Derived-data aggregation method
1	Biographical	Static/single	As is
2	Daily vital data	Time series (daily)	Min, max, mean
3	Braden (4 metrics)	Time series	min, max for each
4	Location/units	Unit names and dates	LOS for each location
5	Periop	Time series	avg, absolute min, absolute max
6	Surgical related data		
	Anesthesia duration	Time series (minutely)	Total duration
	Procedure room stay	Time series (minutely)	Total duration
	Surgical schedule?	Categorical	As is?
	Surgical service?	Categorical	As is?
7	Staffing data	Time series	Mean (RN HPPD and Total HPPD)
8	ASA data (severity of illness)	Time series	Min, max, mean, SD

*American Society of Anesthesiologists (ASA)

Comparing the patients with and without PIs presented a very imbalanced study design, which may obfuscate hidden biases in the results. To address this issue, we introduced a cohort-rebalancing protocol to roughly equalize the sample sizes based on the synthetic minority-sample oversampling technique (SMOTE) [30]. Applying AI techniques such as Random Forests (RF) directly on the raw PI data may be challenging as different patient subgroups may be naturally segregated, such as with surgical service (e.g. orthopedic, trauma, cardiac). For brevity, we don’t show all results; however, we built a global RF model independent of the surgical service, as well as, separate individual service-specific RF-prediction models for each surgical service. Global and service-based model fitting used the corresponding rebalanced cohorts. To simplify the clinical interpretation of the global (hospital-wide) and the service-specific (within surgical service) models, only the 20 most salient features were identified and utilized in the corresponding RF models.

We counted the number of all the services and procedures associated with each hospitalization, including the ones with multiple services. These frequencies were included in a new derived predictive feature whose values were used during the model training phase (Table 2).

**Table 2:** Number of episodes of care by surgical service

Name	Count	Percent
Orthopedics	4081	15.5
Missing	3340	12.7
Neurosurgery	2767	10.5
Urology	2240	8.5
Cardiac	2153	8.2
Trauma	1637	6.2
Thoracic	1489	5.7
Otolaryngology	1255	4.8
Vascular	1128	4.3
Plastics	955	3.6
Colorectal	948	3.6
Transplant	944	3.6
Gynecology	750	2.9
Hepatobiliary	743	2.8
Minimally Invasive Surgery	706	2.7
Oral Surgery	469	1.8
Surgical Oncology	299	1.1
General Surgery Endocrine	251	1.0
Ophthalmology	62	0.2
Dentistry	15	0.1
Podiatry	12	0.0
Anesthesiology	8	0.0
Radiology	4	0.0
Other	1	0.0
Pediatric Cardiac	1	0.0

Cases and features with less than 50% observed values were triaged. The remaining ones were imputed, as needed. Multiple imputation [31–33] was used to generate a computable data object consisting of instances (multiple chains) of the complete dataset with no missing observations.

### AI modeling and analytics

To model the risk profiles of patients, we used model-based and model-free techniques [10, 11, 34–38] and prospectively tested their performance to accurately predict the chance of developing PIs in hospitalization settings. We also examined PI risk globally across the health system, as well as within separate surgical specialty services.

Different machine learning models were fit for each of the following data combinations:

1. All data without Braden metrics and without minority class rebalancing.
2. All data including Braden metrics and without minority class rebalancing.
3. All data without Braden metrics and with minority balancing.
4. All data including Braden metrics and with minority class balancing.

The pragmatics of the clinical applications of an AI app for modeling PI in hospital settings motivates the specific four complementary scenarios investigated in this $2 \times 2$ design. The first factor reflects the availability of the Braden metrics, which are useful, but now always available and resource intensive to compute. The second factor in the design addresses the acute need for rebalancing the data to account for the relatively rare event of developing PI, in general.

**Fig. 2:**
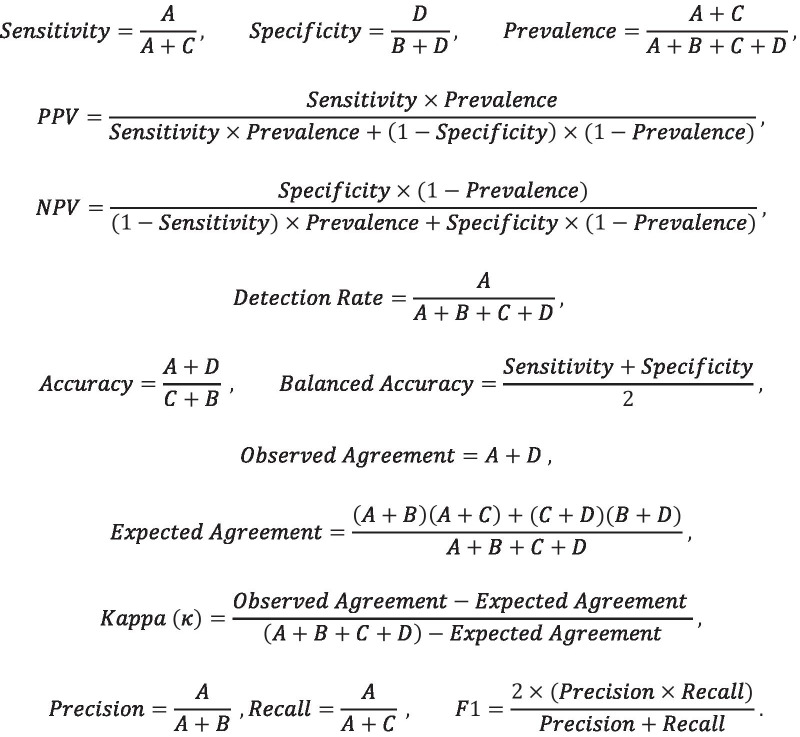
Model evaluation metrics

Model-based PI prediction was accomplished using the generalized linear model (logistic regression) and regularized linear modeling (LASSO) [35, 39–41]. Model-free AI methods included random forests and deep learning [35, 42, 43]. The neural network fit to the data used the keras package. The data was split into training: testing and a sequential network model was fit using the following parameter settings: units = 500, activation = relu or sigmoid, layer dropout rate in the range [0.3, 0.4], layer unit density between 2 and 128, loss function = binary cross-entropy, ADAM optimizer, accuracy metric, epochs = 100, batch size = 10, and validation split = 0.3. Model performance was assessed using metrics shown in Table 3 and Fig. 2.

**Table 3:** Confusion matrix for binary classification

Confusion matrix	Reference	
Predicted	Event	No event
Event	A	B
No event	C	D

## Results

The original dataset included 26,258 cases over the study time period. After removal of cases that didn’t meet the study inclusion criteria (e.g. LOS $\ge 2$ days, and undergoing a surgical procedure, and having staffing data), 18,943 of cases remained. Note that cases were excluded when the event staffing data was not available. Of those, 959 (5.06%) of cases developed a hospital acquired PI during their stay. Average length of stay for the sample was 7.33 days, with a range of 2 to 233. All patients were admitted for a surgical procedure, with the vast majority of patients being admitted under the Orthopedics service, followed by neurosurgery and urology. As noted in Table 2, a large percentage of patients did not have an identified service, thus, they were only included in fitting the global models.

### Data summary

Table 4 shows some of the sample distributions of the stratified cohorts and the complete dataset. The imbalance between PI-positive and PI-negative cases is clear across the strata.

**Table 4:** Main sample-size distributions

Case type	Original data (size)	With LOS ≥ 2	With surgical procedure	Location not in staffing data removed	Intersection final
PI negative	24,928	21,266	21,920	21,601	16,194
PI positive	1330	1142	1015	1137	738
Total cases	26,258	22,408	22,935	22,738	16,932

Table 5 shows the performance of the AI models trained using data with Braden scores. For each model, we show both the results using the raw imbalanced (left) and the post-processed rebalanced (right) datasets.

**Table 5:** Model performance using the Braden scores

AI technique	Original (row, imbalanced) data			Post-processed (rebalanced) cohorts		
	Model			Model		
	
LASSO linear regularized model	Performance of testing data			Model performance of testing data		
	Confusion matrix	Result false	Result true	Confusion matrix	Result false	Result true
	Prediction false	4759	172	Prediction False	4202	38
	Prediction true	14	68	Prediction True	571	202
	Accuracy	96.29%		Accuracy	87.85%	
	95% CI	95.73%	96.80%	95% CI	86.92%	89.74%
	Kappa	0.4079		Kappa	0.3514	
	Sensitivity	0.28333		Sensitivity	0.84167	
	Specificity	0.99707		Specificity	0.88037	
	Prevalence	0.01636		Prevalence	0.15420	

Random forest						
	Confusion matrix	Result false	Result true	Confusion matrix	Result false	Result true
Neural networks	Prediction false	6120	236	Prediction false	6058	225
	Prediction true	99	110	Prediction true	161	121
	Accuracy	94.897%		Accuracy	94.120%	
	Precision	0.5263		Precision	0.4291	
	AUC	0.7252		AUC	0.7134	
	Recall	0.3179		Recall	0.3497	

Table 6 shows the performance of the AI models trained *without* using the Braden metrics. Again, for each model, we show both the results using the raw imbalanced (left) and the post-processed rebalanced (right) data. The LASSO model-based approach resulted in the same model prediction. Clearly the model prediction accuracy using training data without Braden scores is lower compared to their counterparts fit using the Braden scores, Table 5.

**Table 6:** AI model prediction performance using training data without Braden metrics

AI technique	Original (row, imbalanced) data			Post-processed (rebalanced) cohorts		
LASSO linear regularized model	Model			Model		
	
	Performance of testing data			Performance of testing data		
	Confusion matrix	Result false	Result true	Confusion matrix	Result false	Result true
	Prediction False	4775	150	Prediction False	4111	48
	Prediction True	21	67	Prediction True	685	169
	Accuracy	96.59%		Accuracy	85.38%	
	95% CI	96.05%	97.07%	95% CI	84.37%	86.35%
	Kappa	0.425		Kappa	0.265	
	Sensitivity	0.30876		Sensitivity	0.7788	
	Specificity	0.99562		Specificity	0.8571	
	Prevalence	0.04329		Prevalence	0.04329	
Random forest						

Table 7 contrasts the performance of the model-based (regularized linear modeling) and the model-free (random forest) AI predictions. There are notable differences between the two types of AI predictors as well as a clear impact of the availability of Braden scores, which enhance the performance metrics (first column). Rebalancing for the minority (PI) cohort also significantly improves the model prediction accuracy. With or without using the Braden scores and with or without rebalancing, random forest significantly outperforms the linear model-based prediction. The values in the table represent averages of tenfold internal statistical cross-valuation performance.

**Table 7:** Results summary comparing model-based (LASSO) and model-free (RF) prediction on testing data

Metrics	Logistics regression				Random forest			
Design	Using the entire data		Without Braden metrics		Using the entire data		Without Braden metrics	
Balance	Without balancing	With balancing	Without balancing	With balancing	Without balancing	With balancing	Without balancing	With balancing
Accuracy	0.9567	0.86	0.953	0.84	0.985	0.99	0.957	0.994
Kappa	0.3871	0.69	0.302	0.642	0.813	0.979	0.2496	0.987
Sensitivity	0.677	0.767	0.6129	0.72	0.72	1	0.163	1
Specificity	0.963	0.912	0.959	0.9	0.998	0.9855	0.996	0.99
Pos pred val	0.289	0.823	0.217	0.8	0.95	0.973	0.677	0.98
Neg pred val	0.992	0.88	0.993	0.35	0.986	1	0.959	1
Balanced accuracy	0.82	0.83	0.788	0.813	0.859	0.993	0.579	0.996
F1 score	0.40	0.79	0.32	0.76	0.82	0.99	0.26	0.99

## Discussion

Hospital-acquired PIs are difficult to predict in advance, carry significant health burden, and inflate healthcare costs. In this study, we employ innovative data science techniques to predict the likelihood of developing PIs based on available clinical and administrative data. Our results suggest that model-free AI techniques outperform their model-based counterparts in forecasting PI outcomes. This suggests that compared to classical parametric inference, data-driven prediction may provide higher forecasting accuracy due to violations of parametric assumptions (e.g., independence, normality, random sampling, etc.) Sample rebalancing of the EHR training data and inclusion of Braden scores enhance the quality of the models. The improved performance with the Braden score is not surprising as the risk assessment tool has been shown to predict PIs, despite its inability to account for all factors associated with PI risk [4]. Although the performance was improved with inclusion of the Braden scores, the analysis revealed the importance of many other characteristics not included in the Braden tool, as the tool only accounts for moisture, nutrition, sensory perception, friction and shear, mobility, and activity.

Another key finding in this study was the comparison of feature importance among patients in the various surgical services. Unlike previous work, which has shown global predictive results [4, 16] or results based on one specialty group such as cardiac surgery patients [44, 45], we compared feature profiles for patients in over 20 specialty services and found clinically significant differences. This is important going forward because it provides a basis for developing individualized plans of care for the prevention of PI and reducing the health impact of hospital acquired ulcers. For example, the primary risk predictors of orthopedic patients include length of surgery, length of stay, and Braden friction and shear, whereas, patients within the urology service have creatinine value, surgical time, and diastolic blood pressure (during surgery) as primary predictors of PI development. This type of information will make it possible to tailor prevention interventions based on specific type of surgical service. Furthermore, preemptive automatic identification of patients with high-risk of developing PIs in certain surgical services may provide health and economic benefits, in additional to improving patient hospitalization experiences more generally.

As firm supporters of open-science and effective integration of translational science and health analytics education, the authors are sharing the complete source code with synthetic data that illustrate the utilization of this PI forecasting model. This SOCR GitHub Project site (https://github.com/SOCR/PressureInjuryPrediction) includes the end-to-end protocol for the data preprocessing, analytics and visualization used in this manuscript. The sensitivity of the real EHR data used in this study prevented us from sharing potentially personally identifiable information. Hence, we opted to package synthetic data that resembles the real clinical data used in the PI modeling and prediction. We have implemented this technique as a web-app to allow interactive community testing, validation, and engagement.

## Conclusion

Accurate prediction of PI is critical to assure that patients with risk are receiving the nursing care needed to prevent PI development. To date, our understanding of risk has been limited due to limitations in sampling (e.g., one surgical service) and/or methodology (e.g., failure to include all factors predictive of risk). This study is one of the first to use AI techniques with a large, general sample of surgical patients. Findings from this study have identified risk profiles for various surgical services that must be considered when determining prevention intervention strategies to employ. The importance of getting this type of discrete information to the bedside nurse cannot be overstated. To meet this need, we are developing an interactive webapp that implements the RF model to predict PI within specific surgical services or globally for an entire hospital. The app allows interactive forecasting of the probability of acquiring PI in different hospitalization settings using manual data input (one-patient-at-a-time) or in batch model by importing and loading a large number of patient profiles. Thus, in the PIPM prediction model webapp, the concrete cohort of patients can be specified by the research investigator or clinician applying the model to forecast the expected probability of developing a PI during hospitalization based on the individual patient’s data. Discrete data such as this will help the nurse to determine exactly what is needed for each patient, rather than continuing with a more general approach to PI prevention. Such a tailored approach to PI prevention may result in reduced costs (e.g., patients are not receiving care that is unnecessary) and improved outcomes.

## Supplementary Information

**Additional file 1**. Supplementary Dictionary of the compressed variable names used in PIPM.

## Data Availability

Electronic health records (data) are only available to University of Michigan IRBMed-approved researchers. With the IRBMed approval of the study design, no additional administrative permissions were required to access the data. The University of Michigan Data Office can be contacted for more information about data access.
