# Supplementary material for: Modeling and prediction of pressure injury in hospitalized patients using artificial intelligence
Source: BMC Med Inform Decis Mak. 2021 Aug 30;21:253. doi: 10.1186/s12911-021-01608-5 (PMC8406893; doi:10.1186/s12911-021-01608-5)
Supplement: Supplementary file 1 — Additional file 1. Supplementary Dictionary of the compressed variable names used in PIPM. [file 12911_2021_1608_MOESM1_ESM.docx]

**Supplementary Materials**

Dictionary table explicating the compressed variable names into explicit clinically relevant descriptions.

| Label Abbreviation | Description | Construct | Concept |
| --- | --- | --- | --- |
| admissiontype | Admission type | Episode of Care | Admission Type |
| ageinyears | Age | Patient | Age |
| asa_mean.y | ASA Score | Episode of Care | Severity of Illness |
| bmi | BMI | Patient | BMI |
| braden.total.max | Maximum Braden Score | Episode of Care |  |
| braden.total.mean | Mean Braden Score | Episode of Care |  |
| braden.total.min | Minimum Braden Score | Episode of Care |  |
| bradenfrictionsheermin | Minimum Braden Friction and Sheer | Friction & Shear | Skin & Underlying Tissue |
| bradensenspercmin | Minimum Braden Sensory Perception | Pressure | Sensory Perception |
| elix.score_AHRQ | Elixhauser Score AHRQ Method | Episode of Care | Severity of Illness |
| elix.score_Walraven | Elixhauser Score Walraven Method | Episode of Care | Severity of Illness |
| fecaldevice_perc | Percent of days with fecal containment device | Tissue Tolerance | Moisture |
| hr_max | Maximum heart rate | Tissue Tolerance | Perfusion |
| icu_los | Intensive care unit length of stay | Episode of Care | ICU LOS |
| inroom_avg | Multiple procedures average length of time in OR | Episode of Care | Surgical Experience |
| inroom_total | Time in the operating room | Episode of Care | Surgical Experience |
| intubated_perc | Percent of days intubated | Tissue Tolerance | Oxygenation |
| lab_albmax | Maximum albumin | Episode of Care | Labs |
| lab_albmin | Minimum albumin | Episode of Care | Labs |
| lab_creatmax | Maximum creatinine | Episode of Care | Labs |
| lab_creatmin | Minimum creatinine | Episode of Care | Labs |
| lab_glucmax | Maximum glucose | Episode of Care | Labs |
| lab_glucmin | Minimum glucose | Episode of Care | Labs |
| lab_hgbmin | Minimum hemoglobin | Episode of Care | Labs |
| lab_unmax | Maximum urea nitrogen | Episode of Care | Labs |
| lab_unmin | Minimum urea nitrogen | Episode of Care | Labs |
| los | Length of stay | Episode of Care | LOS |
| mean.rn.hppd | Mean RN hours per patient day | Environment | Nurse Staffing |
| mean.total.hppd | Mean total hours per patient day | Environment | Nurse Staffing |
| n_miss_turn_avg | Average number of missed turns | Pressure | Mobility |
| periop_diasbpmax | Maximum intra operative diastolic blood pressure | Tissue Tolerance | Perfusion |
| periop_diasbpmin | Minimum intra operative diastolic blood pressure | Tissue Tolerance | Perfusion |
| periop_hrmin | Minimum intra operative heart rate | Tissue Tolerance | Perfusion |
| periop_rrmax | Maximum intra operative respiratory rate | Tissue Tolerance | Oxygenation |
| periop_rrmin | Minimum intra operative respiratory rate | Tissue Tolerance | Oxygenation |
| periop_sbpmax | Maximum intra operative systolic blood pressure | Tissue Tolerance | Perfusion |
| periop_sbpmin | Minimum intra operative systolic blood pressure | Tissue Tolerance | Perfusion |
| PI_dayfromadmit | Number of days from admission when PI developed | Outcome | PI days from admission |
| pressure_injury_poa | Pressure injury present on admission | Episode of Care | PI on Admission |
| rr_max | Maximum respiratory rate | Tissue Tolerance | Oxygenation |
| SPO2_min | Minimum SPO2 level | Tissue Tolerance | Oxygenation |
| temp_max | Maximum temperature | Tissue Tolerance | Moisture |
| tobaccousedyears | Number of years tobacco used | Patient | Smoking Status |
